# Silo Experience

**Published:** 1882-03

**Authors:** G. Morton

**Affiliations:** Essex, Vt.


### SILO EXPERIENCE.

Almost every day I receive a letter from somebody asking about the wooden silo I built this year, into the hay mow. As all ask the same questions, I will answer, as near as I can, for the benefit of all (from Quebec to Florida I have received letters), and, as I keep no clerk, I find it requires a good deal of time and patience to answer all, as I would do. The best way for my farmer friends to do is to come and see for themselves, cattle and hogs, horses and calves, fed on nothing but ensilage, except *poor* pasturage, since the first of October, with the exception of the hogs, which I feed half meal and bran. The cattle look better than ever before, even when I have fed grain. I find the largest cows need about 100 pounds, and the smaller ones in proportion. As my crop fell awfully short of estimates made in the field, I shall commence feeding some bran to make it hold out. A word about the crop of corn: I planted four different kinds—Blount's, White Prolific, Bailey's, and Southern White. Bailey's turned out far ahead of all the others; many stalks would measure 12 and 14 feet high and weigh 12½ pounds when matured, and it was very easy to figure 100 tons to an acre; but come to get it into the silo, there was a mighty shrinkage—about 140 tons from 8 acres; but I am well satisfied the same land would not cut ¾ of a ton of hay to the acre. The wooden silo I built by simply boarding to barn frame with common, unmatched boards, and filling with sawdust; two sides boarded to studding, leaving a space of about one foot, filled as the other sides with sawdust, the sides well braced, until after filled and weighted, or until done settling, when there is no pressure on the sides. I built this myself in one week; the total expense of cutting and filling 140 tons, outside of my own help, was $42, and the expense of raising, including cutting, did not exceed $100. These are simple facts, and please don't accuse me of exaggeration, for I certainly have no object in trying to mislead my farmer friends or the agricultural papers, from whom I have learned all I know about farming, since the time when, six years ago, after forty-five years at sea, I came up to the old homestead to try farming, hardly knowing how to unrig a horse, as I called it; but with plenty of cider in the cellar; lots of suckers after me for a drink when I went to town; 2-inch auger holes bored in stable floor to let the liquid manure off; but wife's good influence prevailed. I went and heard Moody and tacked ship for blue water, and now I am homeward bound, no running in the dark now for an unknown coast; pilot on board, safe under his guidance. This wooden silo has demonstrated one thing, that a cheap silo answering all purposes can be put into the barn when we want it, alongside of the cows. My stone silo, 20 feet away from barn door only, makes double the work in feeding. I also find I can take my own time filling a silo, by keeping the fodder well trodden down. I put horses on the stone silo, but did not use proper care about the corners, where I found it decayed about six inches.

G. MORTON.

ESSEX, Vt.

---

VICK'S FLORAL GUIDE is one of the best works of its kind in the United States. Send for a copy, and be sure and send for some of Vick's flower and vegetable seeds, as they never fail to grow. We speak from experience. Address James Vick, Rochester, N. Y.

